# The participants’ perspective on facioscapulohumeral muscular dystrophy trials in The Netherlands – A qualitative study

**DOI:** 10.1177/22143602241313117

**Published:** 2025-03-04

**Authors:** Lizan Stinissen, Joost Kools, Sietse Bouma, Emma Lenssen, Eline Sanders, Anke Lanser, Ria de Haas, Baziel GM van Engelen, Wija Oortwijn, Nicol C Voermans

**Affiliations:** 1 Department of Neurology, Donders Institute for Brain, Cognition and Behaviour, Radboud University Medical Center, P.O. Box 9101, 6500 HB, Nijmegen, The Netherlands; 2 Clinical Research Unit, Radboudumc, Nijmegen, The Netherlands; 3 Dutch FSHD patient advocacy group, Wassenaar, The Netherlands; 4 FSHD Europe, Den Haag, The Netherlands; 5 Research Institute for Medical Innovation, Science department IQ Health, Radboud University Medical Center, P.O. Box 9101, 6500 HB, Nijmegen, The Netherlands

**Keywords:** neuromuscular diseases, qualitative research, patient participation, clinical trials

## Abstract

**Background:**

Facioscapulohumeral muscular dystrophy (FSHD) is a hereditary muscle disease without an available cure. The first trials with potentially disease-modifying therapies have started, including a phase ll open-label study and a phase lll double-blind randomized placebo-controlled trial assessing the safety and efficacy of losmapimod. Having a more in-depth understanding of the patient's experience of these trials will further enhance the design and recruitment of future trials.

**Objective:**

To explore the motivation, expectations, concerns, and experiences of FSHD patients in the first clinical trials in the Netherlands resulting in recommendations for future trials.

**Methods:**

Semi-structured interviews with participants of phase II and III losmapimod trials were conducted. The interview guide was based on previous conducted literature reviews and consultation of a patient representative. Participants were selected through convenience sampling. Four main themes were discussed: motivation for participation, expectations regarding study drug and trial visits, trial participation experience, and recommendations for future trials. The interviews were transcribed, anonymized, and analyzed using Atlas.ti version 23.1.1 using a deductive approach.

**Results:**

Thirteen participants were interviewed; six phase II participants and seven phase III participants. The primary motivations to participate concerned altruistic motives, contribute to science or improve their own health status. The participants had realistic expectations of the effect of the study drug before trial participation. Overall, participants were positive about their trial participation. Specifically, the personal and transparent communication within a trusting and dedicated trial team was appreciated. The phase III participants reported a higher than expected psychological burden on participating in a placebo-controlled trial. Recommendations consisted of more frequent updates on the overall progress and results of the trials.

**Conclusions:**

This study presents the participants’ perspective on FSHD trials, providing important key findings for future clinical trial design, study site practices and patient education.

## Introduction

Facioscapulohumeral muscular dystrophy (FSHD) is a hereditary muscle disease affecting approximately 1:2000–10,000 persons.^1,2^ Patients with FSHD usually experience weakness of the face, shoulder and upper extremity skeletal muscles initially, followed by weakness of the leg, hip girdle and trunk muscles.^3^ Notably, approximately 20% of the patients eventually become wheelchair dependent.^4^ The contemporary management of the disease consists of a multidisciplinary approach to reduce the symptoms of FSHD, e.g., by improving muscle endurance and reducing pain and fatigue.^5^ Currently, no pharmaceutical disease-modifying treatment for FSHD exists.

From 2010 onwards, the understanding of the underlying genetic and pathophysiological mechanism of FSHD has increased.^6^ This allows for the development of drugs that specifically target FSHD pathways. The first trials with possible new disease-modifying therapies have already started and the number of trials is expected to increase in the coming years, with over twenty companies developing new interventions.^7^ Two clinical trials were recently conducted at the Radboud University Medical Center (Radboudumc) in Nijmegen, The Netherlands: a phase II single-center, open-label study and a phase III multi-center placebo-controlled study of losmapimod.^8,9^

To enhance trial design, several steps have been taken to improve clinical trial readiness in the recent years: natural history studies were conducted, new clinical outcome measures were developed, and (inter)national patient registries were set-up.^10,11^ Furthermore, several international collaborations were initiated such as FSHD Europe, the FSHD Clinical Trial Research Network and Project Mercury. On the contrary, evaluation of the trials from a patient's perspective is lacking while it could provide useful insights to improve future trial design.^12^ For example, we evaluated the experienced burden of muscle biopsies performed in the phase II losmapimod trial and other studies using a quantitative survey. The pain experienced as a consequence of the biopsies was higher than we initially expected. We therefore advised for more careful consideration when adding muscle biopsies to a study protocol, minimize the frequency and explore more patient-friendly methods.^13^ In addition, we performed a scoping review regarding the experience of clinical study and trial participation in rare diseases. It describes the barriers, facilitators and lessons learned of patients with Duchenne muscular dystrophy and SMA in clinical trials.^14^ Unfortunately, we could not find studies on the experience of FSHD patients. Therefore, we aimed to explore the motivation, expectations, concerns, and experiences of FSHD patients in a clinical trial. The insights from this study are expected to improve future clinical trial design, study site practices and patient education.

## Methods

### Design and data collection

This study followed a phenomenological, interpretive design using semi-structured in-depth interviews with participants from two FSHD drug trials which were ongoing at the time of the interviews at the Radboudumc.^8,9^ Participants were informed verbally and in writing regarding the study's objective, methodology, and procedures of data storage. A junior researcher not involved in the execution of the trials (SB) performed semi-structured interviews with a duration of approximately one hour. The interviews were either performed on-site (generally combined with a study visit) or online via Microsoft Teams, depending on the preference of the participants. All interviews were recorded and transcribed by the same junior researcher (SB). Findings are reported according to the Consolidated Criteria for Reporting Qualitative Research (COREQ), supplemental data 1.^15^

### Study population and recruitment

Of the 27 participants in the losmapimod trials, 20 participants were invited for an in-depth interview. Due to time constraints, the participants were selected through convenience sampling by SB and JK, based on availability during working hours and already scheduled study visits. The invitees concerned 9 participants of the phase ll trial and 11 participants of the phase lll trial. These were patients with genetically confirmed FSHD, aged 18–65 years and a clinical severity score of 4–8 (i.e., weakness in upper and lower extremities while still being able to mobilize with possibly assistant devices). At the time of the interviews (June-July 2023), the phase II trial was running for approximately four years, while the phase III participants were enrolled for approximately 4–24 weeks. We aimed for an even distribution of phase II and III trial participants and sexes, yet with different ages. All patients were invited for participation in the study by SB through e-mail. When patients were interested in participation, they were contacted by telephone for further explanation and for scheduling the interview. Participants were included until data saturation was reached.

### Phase II and phase III losmapimod studies

Losmapimod is a repurposed drug with a favorable safety profile tested in over 3500 individuals.^16^ It has been tested as a potential disease-modifying therapy in FSHD after the discovery that it could reduce DUX4 expression in human FSHD myoblasts.^17^ Recently, the results of the phase III trial turned out to be negative and the program was halted.^18^ At the time of the interviews, the trials were still ongoing. The phase II trial had a more intensive design compared to the phase III trial.

The phase II losmapimod study was an open-label, single center study aiming to assess long-term safety and tolerability of losmapimod treatment in FSHD patients.^8^ Secondary and exploratory outcomes included biomarkers in blood and muscle tissue (collected through two muscle biopsies), changes in imaging biomarkers (using MRI and muscle ultrasound) and changes in several strength and functional outcome assessments, such as the 6-min walk test and manual muscle testing. Hospital visit duration ranged from approximately 3–6 h. The baseline measurement consisted of 3 visits over 8 weeks, followed by visits every 12 weeks. After one year, participants had the option to enroll in an extension phase, continuing the visit schedule but with a reduced number of clinical outcome assessments.

The phase III losmapimod study was a randomized placebo-controlled multicenter study aiming to assess efficacy of the drug using the upper extremity function as the primary outcome measure.^9^ After a screening visit, participants were randomized into the placebo or treatment group. After randomization, visits took place every 12 weeks. Hospital visit duration ranged from 1.5–3 h. After 48 weeks of treatment, participants had the option to enroll into the open-label extension phase.

In this qualitative study, we purposefully included participants from both studies as they could provide different perspectives on trial participation, due to the differences in the trial designs. It is not our aim to compare the experience of participants regarding the two clinical trials, but notable observations between the trials will be reported.

### Interview guide

The interview guide was developed based on several sources describing the concept of conducting in-depth interviews, previous conducted literature reviews regarding patient experiences, clinical (trial) experience and consultation of the patient representative and chairman of the FSHD Advocacy Group (AL).^12,19–21^ The interviews started with exploring the motivations for participation. Secondly, the expectations regarding participation, drug efficacy and risk of adverse events were discussed. Thirdly, we inquired about the communication related to the trial, including the method of recruitment, informed consent, scheduling of visits, instructions during the trial period and communication from the sponsor about study progression and results. Furthermore, specific topics pertaining to a placebo-controlled randomized trial (e.g., the uncertainty regarding the received treatment) was discussed with phase III participants. Lastly, the trust and hope in clinical trials were discussed as well as recommendations for future trials. The full interview guide is available in supplemental data 2.

### Ethical considerations

The study protocol was approved by the Radboudumc research ethics committee (file number 2023-16354). This ensures that the study is carried out in accordance with the applicable legislation (Medical Research involving Human Subjects Act and Medical Treatment Contracts Act).

### Data analysis

Transcripts of the interviews were made by a native speaker (SB) and analyzed with Atlas.ti version 23.1.1 using a framework analysis. All participants were pseudo-anonymized in the transcripts, using numbers to indicate the participants. The transcripts were independently coded by two researchers (SB, LS) using a deductive approach to generate codes, based on the theoretical framework used to develop the interview guide. We systematically coded the subsequent interview transcripts until data saturation was reached. Subsequently, codes were divided into main themes and subthemes, as discussed by two researchers (JK, LS).^22,23^ The transcripts and interpretation of the findings were not discussed or shared with the participants.

### Reflexivity statement

Three researchers (SB, LS, JK) were involved in performing and analyzing the interviews. SB is a white male in his mid-twenties who performed this study as part of his final internship for his master's degree in Science in Society. He is familiar with neuromuscular diseases as he performed a previous internship on respiratory characteristics of Dutch individuals with a diagnosis of centronuclear myopathy at the Neurology department of Radboudumc. He attended several clinics with neuromuscular patients. He had no relation with any of the participants nor was he involved in the execution of the losmapimod trials. He was trained by a senior social scientist experienced in qualitative research. LS is a white female in her mid-twenties working at the Radboudumc as a PhD candidate after receiving her Biomedical Sciences master's degree. She has prior experience in conducting qualitative studies in neuromuscular disorders, but was not involved with FSHD patients or the losmapimod trials prior to this study. JK is a white male in his early thirties working as a trial physician for over five years conducting the phase II and III trial while finishing his PhD candidacy on trials in FSHD.

## Results

Of the 20 participants that were invited, thirteen participants completed an interview. One participant did not want to participate because she felt she could not reliably contribute due to the low number of weeks she was enrolled in the trial. Six participants did not respond to the initial invitation. Follow-up was not required since data saturation had been reached after 13 participants had been interviewed. All of the interviewees fully completed the respective trial. During one interview, the spouse of the participants was present. Patient characteristics are shown in Table 1.

**Table 1.** Participant characteristics.

Participant	Sex	Current age (years)	Age at diagnosis (years)	Phase trial (weeks in trial)	Social status	Highest education level	Work status (hours per week)	Location interview (Radboudumc/online)
1	F	55	33	II	2	1	9	Online
2	F	48	28	III (12)	2	2	32	Online
3	M	57	12	III (24)	2	1	Not anymore	Online
4	M	27	14	II	2	1	32	Online
5	F	42	19	III (24)	2	3	15	Radboudumc
6	F	52	32	III (4)	2	4	Not anymore	Radboudumc
7	M	55	32	III (24)	2	3	<40	Radboudumc
8	M	57	40	II	2	2	40	Online
9	M	51	26	III (24)	1	1	32	Online
10	F	62	60	III (24)	2	1	32	Online
11	M	37	16	II	2	2	Not anymore	Online
12	F	53	43	II	2	1	24	Radboudumc
13	M	61	33	II	2	1	Not anymore	Radboudumc

F = female, M = male. Social status reaches from 1 to 2 (1 = living alone; 2 = living with partner/family members). Highest education level reaches from 1 to 4 (1 = vocational education; 2 = higher professional education; 3 = university; 4 = PhD).

**Figure 1.**
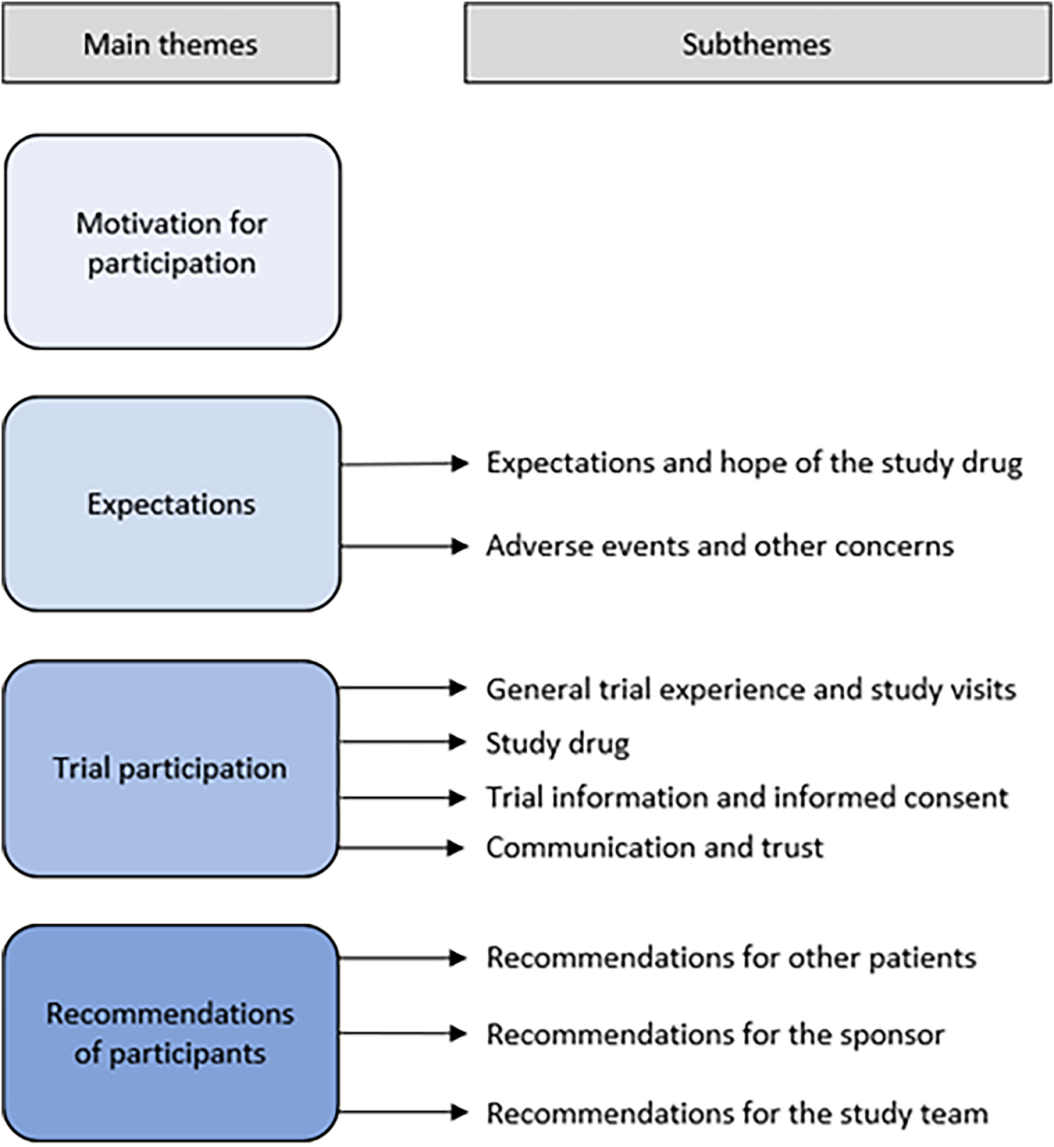
Main themes and subthemes identified from interview findings.

A total of 718 quotes were identified, divided over the following main themes: Motivation (69 quotes), Expectations (136 quotes), Trial participation (461 quotes), and Recommendations (52 quotes) (Supplemental Table 1). Trial participation is the most extensively discussed theme. This theme also had the most identified subthemes, especially Communication and trust, General trial experience, and study visits were extensively discussed (163 quotes each). The frequency of quotes per patient ranged from a minimum of 48 to a maximum of 62 quotes. An overview of the main themes and identified subthemes are presented in Figure 1.

### Motivation for participation

A wide range of motivations for participating in the trials were reported (69 quotes). Illustrative quotes are presented in Table 2. Almost three quarters of the participants had no doubts in deciding to participate. The doubts of the other participants that were mentioned concerned questions regarding the possibility of continuing the drug after the trial was finished, possible side effects mentioned in the informed consent form, the time investment and the invasiveness of the study visits. More than three quarters of the participants participated from an altruistic perspective: to help the development of a therapy for their children or the new generation of FSHD patients. Almost half of them wanted to contribute to scientific research in FSHD, acknowledging that these kinds of studies are only successful if patients are willing to participate. The possibility of being enrolled in the placebo-arm was not considered as a barrier for participation by the Phase lll participants. Participants were aware there was a 50% chance of receiving either the study drug or the placebo. They regarded this as something they could accept, which was reinforced by the opportunity of enrolling in the open-label extension study. The certainty of receiving losmapimod after 48 weeks in the open-label extension improved their motivation for participation Phase III participants additionally reported that they were participating to potentially improve their own disease state and to get early access to a possible new therapy. Besides the primary motivations, more than a quarter of the participants felt a responsibility to participate in trials because of the rareness of the disease. Furthermore, participation gave them a sense of agency. Suffering from FSHD meant a continuous process of losing muscle function, either invoking sadness or fear for the future. Participating in a drug trial gave participants the opportunity to actively try to combat the disease, regardless of actual drug efficacy.

**Table 2.** Illustrative quotes on motivation for participation.

Subthemes	Quotes
Motivation	*“Yes, self-interest. Yes. That's really the most substantial, self-interest, and I hope that I benefit from it.” P2* “*And yes, it was obviously like this: if you received the placebo, you’d get the real medication in the end. So, either way, you’d receive the medication sooner than if you didn’t participate.” P9* “*Obviously for myself as well. I really want it to work for me too, but that's not reason number 1 for me. Reason number 1 is truly for my children and others. That there will be a medicine in the future that slows it down.” P8*

### Expectations

In total, 136 quotes were identified in this theme, with illustrative quotes provided in Table 3. The expectations were divided into two subthemes: Expectations and hope of the study drug (101 quotes), and Adverse events and other concerns (35 quotes).

**Table 3.** Illustrative quotes on expectations.

Subthemes	Quotes
From/of study drug	*“I actually knew beforehand that there would be no medicine that would miraculously cure the FSHD. I mean, it's something in your DNA. If you could cure it with a pill, that would be very miraculous, but there's still that hope that it would slow it down.” P1* “*You shouldn’t have expectations of medications; you should go into it blank.” P10*
Adverse events and other concerns	*“And in that sense, the doubt that was there was something like, ‘Am I going to be able to handle that?’ I have quite a busy job and, well, perhaps a bit less energy than the average person, due to FSHD.” P2* “*Yes, of course. Because it's a medicine that's already been tested before, I was like, ‘Yes, the chance that something could go wrong is relatively small. For example, as compared to a genetic test.’ I would have to think about that a bit more carefully.” P6*

#### Expectations and hope of the study drug

Participants mentioned a clear distinction between their expectations and hopes regarding drug efficacy. In general, participants had realistic expectations about the study drug. They understood the experimental nature and unknown efficacy. The phase III participants understood the necessity for a placebo-arm. More than half of the participants did not expect a cure, but a halt or reduction of the disease progression. Because of the slowness of the disease progression they did not expect to notice any change themselves in disease state during the studies.

Both the preclinical data and the results of the phase II study played a major role in setting these expectations and hopes while simultaneously increasing the motivation for participation. This information was gathered through at least one of the following channels: trial-specific patient information forms, webinars organized by the Sponsor or Dutch patient advocacy group, patient conferences organized by the Dutch patient advocacy group, and conversations with trial physician JK. Besides the losmapimod studies, the increasing interest in drug development for FSHD by several pharmaceutical companies gave participants hope for a disease-modifying therapy in the future. the hope that losmapimod could be the first disease-modifying treatment was reinforced in phase ll participants due to the start of the Phase lll trial.

#### Adverse events and other concerns

Expectations on possible adverse events of the drug were based on the patient information provided for the study. In general, participants had no concerns pertaining to the adverse events, as the patient information included information about losmapimod's known, favorable safety profile in healthy individuals. The possibility on drug efficacy study effects outweighed the possibility on adverse events for deciding to participate in the trials.

Almost a quarter of the participants did report concerns about the time investment before starting with the study. Especially the participants with a full-time job mentioned they had concerns about combining their daily life activities with the required study visits. An additional concern mentioned was about the possible psychological effect if the drug turned out to be inefficacious, causing the sponsor to possibly stop the trial.

### Trial participation

Trial participation was the most common theme, with a total of 461 quotes, with illustrative quotes presented in Table 4. This includes the following subthemes; General trial experience and study visits (163 quotes); Study drug (55 quotes); Trial information and informed consent (80 quotes); Communication and trust (163 quotes). Overall, patients had a positive experience regarding the trial participation within the Radboudumc.

**Table 4.** Illustrative quotes on trial participation.

Subthemes	Quotes
General experience and study visits	*“I actually expected it to be more intense, but yeah, I got through all the tests in a few hours. And then the MRI is once every three months. Yeah, I don’t think it's actually that bad.” P8* “*But here, I feel exactly as if I’m the only one doing the study, so all the focus is on me when I’m here… but at the other place, you also came together with other participants from other studies, and that felt different. There, I felt more like a number. Yes, and here, that's not the case.” P7*
Study drug	*“If you basically feel no side effects at all after a few weeks, then the anxiety will go away as well. After the first two days, it was already a lot less, but in the beginning, it's still somewhat nerve-wracking when taking the first pill. Maybe you won’t feel well or whatever, or maybe you suddenly feel deterioration, or whatever side effect.” P11* “*I’m a very down-to-earth person, but it still surprises me how much influence whether or not you get a placebo has on your thoughts. Especially in the first few months. Now I’m at peace with it, but I used to worry about it. Yeah, am I feeling something? I’m suffering from that now. Is it mainly because of the disease, or is it because of the medication? There's a bit of uncertainty involved when you’re participating.” P8*
Trial information and informed consent	*“Yes, they’re about the same every time, communication went well… I think I’ve asked all the questions I wanted to ask, but it just felt right.” P5* “*But indeed, during that webinar, I thought, ‘Oh, hey, they apparently see something positive here, otherwise they wouldn’t continue.” P1*
Communication and trust	*“Yes, nice, pleasant people. Appointments are fast and efficient. You’re approached personally; you don’t feel like a number. I can’t say anything other than that it's going very well and feels good.” P8* “*So, in that respect, I find it very pleasant and also very important that there was really just one person who was there all the time.” P10* “*And I do occasionally want some information on how other people are dealing with it, but often, those people are worse off than I am and then, well, you actually don’t want to be confronted with all the troubles of other people.” P9*

#### General trial experience and study visits

All participants expressed gratitude for being able to participate in the trial. Especially the phase III participants expressed this, as they were aware of the huge interest in the trial and the fact that not every patient could be included. Overall, it was appreciated that they were the only participant present during visits, receiving full attention from the study team. The guidance for each test was well received, although sometimes reported as too much repetition at subsequent visits. Due to the clearly communicated schedules, patients did not experience large surprises during the studies. Overall, participants of the phase II study mentioned a high intensity of the visits during the first year, sometimes having underestimated the toll it would take on their body, resulting in fatigue and/or muscle aches the day after study visits. On the contrary, phase III participants experienced the visits as less intensive than expected. In general, the muscle biopsies (phase II) and MRI scans(phase II and III) were reported as the most burdensome assessments due to pain caused by the biopsy procedure or prolonged supine position. Some of the functional tests were also considered burdensome in severely affected patients due to the exertion on the body (e.g., the six-minute walking test for a participant with severely affected leg muscles).

#### Study drug

Unlike in daily life, almost a quarter of the participants mentioned to be confronted with their disease state and physical limitations every time they took a study pill, reporting this as a drawback for participating. This was most pronounced for phase III participants because of the possibility of receiving a placebo. During the trial, almost half of the participants seemed to have acquired an intense focus on physical functionality, wondering if a certain sensation could be an adverse event suggesting treatment with losmapimod, or experiencing disease progression suggesting taking placebo. Two participants hoped they were being treated with placebo as they did not experience any efficacy at the time. Regardless of phase II or III, participants reported on the difficulty of detecting any change over time due to the slow progression of the disease. This caused uncertainty on the efficacy of the drug and made it hard to reliably complete questionnaires about the disease progression.

#### Trial information and informed consent

The Dutch FSHD-registry was the most common channel through which participants had received information on the trials. Other channels were the patient advocacy group, treating physician, family, social media or during other study visits. All participants understood the patient information form, although two participants found the length of the letter daunting. Sometimes, participants also searched on the internet for additional information regarding the study, which was hard to find. More than three quarters of the participants shared the information with their partner or close family. There were some uncertainties after reading the information, which were addressed by the study physician via phone, email or during the study visits. This mainly concerned some general questions regarding the placebo, the trial design and reimbursement. On top of that, two participants experienced the language and process of signing informed consent as too formal and therefore difficult to fully comprehend or a hassle to complete for every new amendment.

#### Communication and trust

All participants reported that both the trial physician and study nurses were easily accessible for questions during the study. They felt the communication was transparent, personal, and non-hierarchical. In general, participants expressed appreciation about the study team setting realistic expectations for both the study drug and visits. Knowing what to expect, via a per visit schedule and a yearly schedule, was of great value for the participants. Due to a small, dedicated study team, participants always encountered the same physician and nurses, reportedly increasing the trust in the study team over the course of many study visits. The need for contact with other study participants differed. The study visits were scheduled individually with the intention of ensuring that patients do not come into contact with each other. More than half of the participants did not want to meet other patients, mainly because they were anxious to meet more severely affected patients, confronting them of what could be their future. More than a quarter of the participants did express the desire to share their trial experience with other participants, mostly wondering if other participants experienced any efficacy from the drug. Some participants did not have a desire to meet other participants, but would be willing to meet them if they could support other participants this way.

### Recommendations for future trials

A total of 52 quotes were identified regarding the theme on Recommendations, with illustrative quotes in Table 5. This is divided over the subthemes Recommendations for other patients (13 quotes), Recommendations for the sponsor (12 quotes), and Recommendations for the study team (27 quotes). Although the participants were positive about the communication during the trial, most of the recommendations involved improving the communication.

**Table 5.** Illustrative quotes on recommendations.

Subthemes	Quotes
For other patients	*“For me, this was a relatively individual consideration, and I think it would be for everyone. But without research, nothing will ever happen. So, yes, if you get the chance, go for it.” P1* “*There are obviously quite a few risks involved, so I would definitely say that everyone should decide that for themselves. Of course, I invite everyone to help in this, whenever they can. But I wouldn’t want to force anyone to do this; they really need to weigh the risks and time and such for themselves.” P11*
For the sponsor	*“Yeah, I don’t expect there to be a special presentation for 14 people. But it could simply be a newsletter distributed by the hospital among those participating here, that's also possible. It's not that you have to have direct contact, but I’d really appreciate it if I could get have insight into the status of the research from the pharmaceutical company.” P8* “*Well, maybe you can sign up for some kind of newsletter, or whatever, and then you can choose for yourself whether you want to receive it or not. But that at least some results could be shared.” P3*
For the study team	*“Yes, of course, the more communication, the better, even if it's just a small message that's published in the muscular disorder newsletter or posted online or shared on Facebook. You see, if there would be a brief update posted once a month, that would be very nice for the research. It doesn’t have to be extensive. Or, just to mention something, if there's a new medication coming out and they’re looking for new people.” P6* “*Yes, and that's often missing in the processes. That you don’t really know when a certain period has ended. Exactly how long will it continue? Am I the last one? Or when can you expect the results? These may also be things that you probably don’t know right away at the beginning. But just a brief update?” P5*

#### Recommendations for other patients

None of the participants would advise against participating in a trial. Almost half of the participants would recommend it, describing it as a unique and pleasant experience and acknowledging the need for sufficient participants in rare disease trials. They also felt that they had contributed to scientific progress. Furthermore, almost half of the participants mentioned that the decision to participate is a personal decision depending on availability (e.g., full-time job), disease severity and travel time to the clinic.

#### Recommendations for the sponsor

Most of the recommendations were about the lack of communication during the trial as well as the lack of sharing study data by the sponsor. First, more frequent updates on the progress and results of the study would be greatly appreciated. Participants suggested the use of a recurrent newsletter from the sponsor. Furthermore, although most participants knew that study data could not be shared ahead of time, they would appreciate a personal data report on their disease progression. Lastly, communication regarding the process of reimbursement of travel costs and overnight stays was not entirely clear and could be improved.

#### Recommendations for the study team

Even though participants were overall positive regarding the communication with the study team, more updates from the study team would be appreciated. This mainly included more communication on the overall planning and updates concerning the progress of the study.

## Discussion

**Figure 2.**
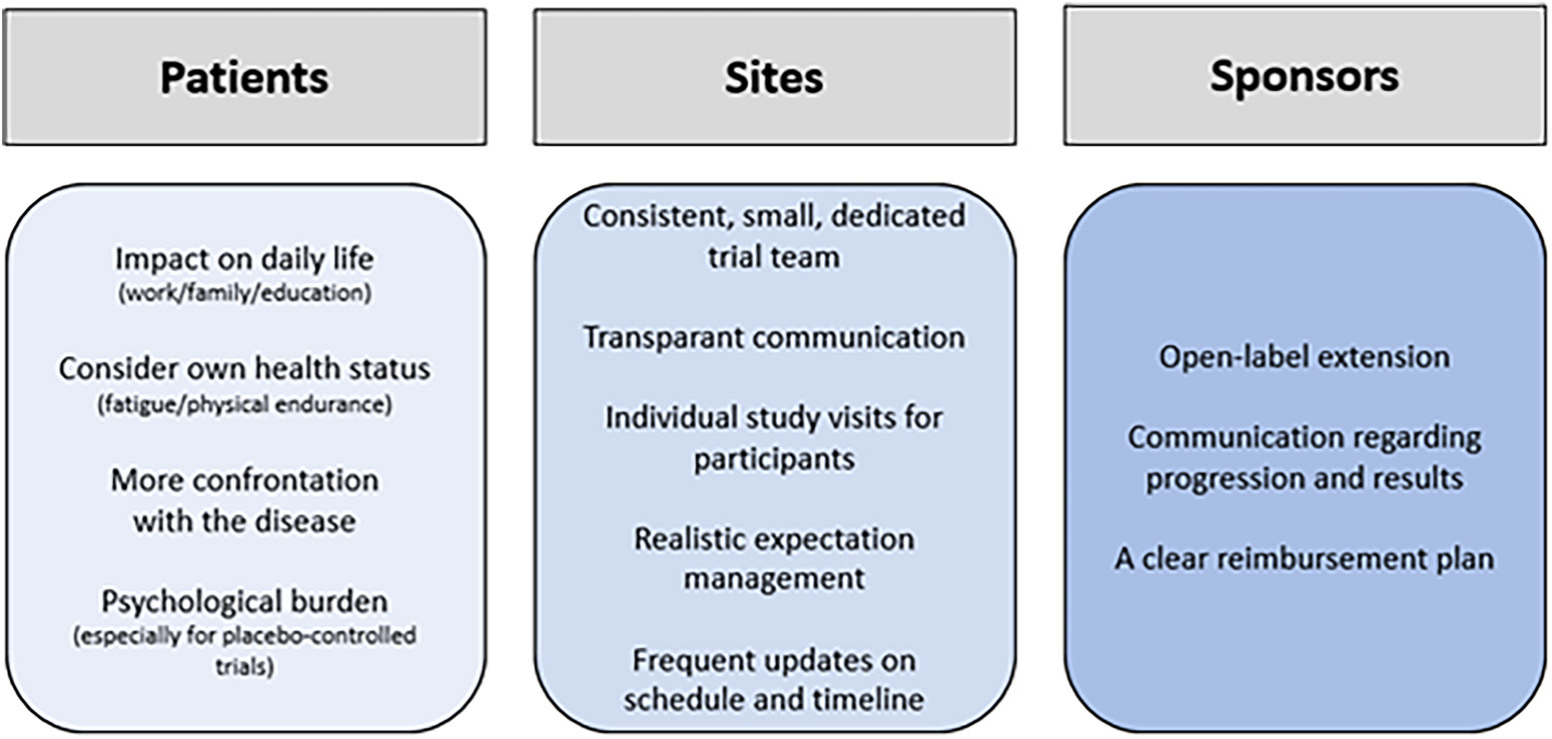
Key points.

This qualitative study was initiated to gain insight in the trial experience of FSHD patients to improve future trial design, including site practices and patient education. The motivations for trial participation ranged from altruism to hope for improved health status. The participants’ expectations regarding study drug efficacy were realistic (stabilizing muscle strength and function), while hope for a potentially higher efficacy was also reported. Trial participation was seen as a positive experience, largely driven by the personalized approach of the study team. Recommendations from the participants included more frequent updates on the progress and results of the trials. Participants acknowledged that the decision about trial participation requires personal considerations, but none of them advised against participation in a clinical trial. A summary of the most important key points are presented in Figure 2. We will discuss these main findings below.

Although this study was performed specifically in FSHD patients, we hypothesize that most of the recommendations are also applicable for other neuromuscular disease trials, perhaps even for all rare disease trials. The findings in this paper are supported by our recent scoping review on trial participation in rare diseases and our qualitative study on trial experience of centronuclear myopathies patients.^12,14^ The most important facilitators of trial participation were hope for improving the participants’ health, altruistic motivations, and gaining a better understanding of the disease. Barriers included unknown efficacy and side effects, the chance of being treated with a placebo, and logistical and financial challenges (e.g., travel time, missed school- or workdays, out-of-pocket expenses). The possibility of receiving a placebo can be a barrier for participants, resulting in a patient's preference for clinical trials without a placebo-arm. In the case of the phase III losmapimod trial, this barrier was overcome by allowing participation in an open-label extension phase after completing the randomized trial phase. If possible, including an open-label extension phase or compassionate use program will benefit the recruitment process of future trials. Lastly, another study found that patients who participated in clinical trials were more self-efficacious, less anxious and reported a higher level of social support compared to patients who declined participation.^24^ Although elucidating the full extent how these characteristics influence participation and drop-out requires more research, awareness of these characteristics might enable healthcare providers and trial sites to improve recruitment rates and reduce drop-outs.

While we hypothesize that the majority of the recommendation are translatable, participants might experience future trials differently depending on the study protocol, study population and the country in which the trial will be conducted. We observed that phase II participants experienced the trial as a whole more burdensome in comparison to phase III participants. This was expected, as the phase II visits were of longer duration and included significantly more muscle strength and functional assessments compared to visits of the phase III trial. Still, as opposed to other neuromuscular disease trials, the frequency and intensity of the visits of the losmapimod trials can be considered relatively low. The losmapimod studies required one visit every twelve weeks after a slightly more intensive study start compared to, for example, a myotonic dystrophy study which requires a visit every four weeks (NCT05481879) or a Duchenne trial requiring weekly on-site intravenous infusion (NCT04060199).^8,9,25,26^

The losmapimod trials included mostly independent adult patients, which will be different from pediatric patients who require the support or presence of their parents or other caregivers to participate in a trial. Including care-dependent patients will increase the difficulty of trial participation significantly. For example, in the centronuclear myopathies trial parents often had to take time off from work to support their child during a trial visit.^12^ Additional qualitative studies in other neuromuscular disorders will be important to make disease specific recommendations.

Travel time and overnight stays were reported barriers to participation in our scoping review, but not often mentioned by the participants of this study. This is most likely caused by the geographical region of this study (The Netherlands and Belgium); the mean travel time to the site was approximately 90 min and an overnight stay was optional based on the participant's preference. The travel time in larger countries will be significantly longer, possibly even include travel by airplane.

Both in this study and the study on trial experiences in centronuclear myopathies, participants reported to participate for their own health benefit.^12^ While we want to refrain from valuing the motivations for participating in trials, participating for personal health benefit warrants caution. As the efficacy of study drugs are still unknown, participating in trials for personal health benefit is a result of therapeutic misconception. A quantitative questionnaire study in participants with degenerative ataxia also reported on the misconceptions of the efficacy of an experimental treatment.^27^ This suggests that therapeutic misconceptions are a widespread issue in rare diseases without an available treatment. Arguably, therapeutic misconceptions may interfere with informed consent and could therefore pose an ethical problem. The nuance of an experimental treatment compared to standard clinical care apparently seem to require additional attention when informing patients about a trial. An exemplary quote in our previous study was “*A trial is not a treatment*”.^12^ Assessing the expectations and motivations of the participants before signing informed consent, for example during a pre-screening interview, should therefore become standard practice. Additionally, patient education on trials should emphasize the difference between standard clinical care and receiving an experimental treatment.

Accurate patient education also serves other purposes. Patients need to be prepared on what trial participation entails to prevent dropouts or unexpected experiences. For example, patients reported a high psychological burden (e.g., negative confrontations with their disease, stress due to uncertainty of treatment arm) of participating in a trial, with the burden being higher in the placebo-controlled trial, or the discomfort of lying in a supine position in the MRI machine for over an hour. They would have liked to have been informed beforehand about these experiences. Patient advocacy groups could play an important role in ensuring that the different forms of patient education matches the patients’ expectations, is clear and understandable and is easily accessible. For example, the Dutch Muscle Disease Foundation (Spierziekten Nederland) organizes a yearly patient conference to update patients. Patients benefit from the scientific and clinical information and enjoy the social aspect of sharing experiences with other patients. We think that additional (online) patient education events for new trials would be of great added value, as webinars can reach more patients and are rewatchable. The use of patient navigators (i.e., patients with experience who guide new patients) provide a more personalized approach, but might be more difficult to arrange.^28^

This study highlighted the patients’ preferred interactions with the trial site. This can support site preparations for new clinical trials. The use of a small, dedicated trial team was greatly appreciated by all participants. It allowed for clear communication, personalized care, and building up trust and loyalty, which were all important aspects in maintaining motivation throughout the trial. Using a dedicated trial team also enables clear separation between trial and regular care visits, which is beneficial for both the researcher and the patient. Separation of a trial and care physician may reduce the chance on investigator bias, especially regarding the clinician's impression of change of the disease state. From an ethical perspective, the argument can be made that both the screening and the informed consent procedure will be influenced by the loyalty of the patient and treating physician. Still, we argue that it is beneficial to perform trials in centers with experienced treating physicians in the case of (unexpected) adverse events and to ensure that the trial team has sufficient knowledge about the disease.

Communications from the sponsor was the part that received the most recommendations. The participants would have appreciated more frequent updates about the overall progression of the trials, drug efficacy and, if possible, their own data. Trials in FSHD, and possibly other neuromuscular disorders, might require a duration of multiple years before efficacy is assessed (e.g., the phase II losmapimod trials had a duration of five years). Keeping participants actively involved during these long trials by frequent updates will help maintain the participants’ motivation and in turn reduce the number of dropouts. To protect the privacy of patients and reduce the influence of the sponsor on the trials, sponsors are not allowed to have direct contact with participants. It is therefore important to identify the correct channels for informing the participants, while adhering to good clinical practice guidelines. Most of the participants suggested using a newsletter, which could be sent from sponsor to the investigators and subsequently be distributed to the participants. Another solution could be the use of a dedicated website as used for the FSHD Fortitude trial, which removes the need for additional channels to reach the participants.^29^ Regardless of the method, a clear communication plan from the sponsor and sites would be highly appreciated by participants and should be in place before the start of the trial.

This is the first qualitative study exploring the experience of clinical trial participation in FSHD patients, resulting in several key points to improve trial readiness. It is important to interpret these results with the strengths and limitations of this study in mind. The interviews in this study were purposely performed by an independent researcher with the notion that the participants might be less inclined to give socially acceptable answers. Nevertheless, participants might have been reluctant to give negative answers because the trials were still ongoing. An aggregate approach in the data analysis was chosen to increase the generalizability of the results. Both phase II and phase III participants were included, which gave insights in the different motivations of participants per trial phase and the additional psychological burden of a placebo-controlled trial. The phase II trial was ongoing for more than four years at the time of this study, which gave better insight into the long-term experiences, but might have introduced recall bias. Although the recruitment of participants continued until data saturation was reached, we cannot rule out that selection bias might be present due to convenience sampling. Lastly, we did not validate our analysis and interpretation of the interviews by asking the participants to review our interpretation. This is a missed opportunity and it is strongly recommended to include in future qualitative studies.

With the expected increase of clinical trials in neuromuscular diseases, studies on patient's views and experience will become essential to inform the design and implementation of future trials. This study was performed relatively late in the losmapimod trial development process, diminishing the possibility of direct implementation. Therefore, we suggest that future patient's views and experience studies should be performed as early in the drug development process as possible. We suggest that prospective trial evaluation from the patient's perspective, quantitative or qualitative, should become an integral part of every future trial. The use of the patient involvement matrix can ensure the incorporation of the patient's perspective in future study/trial design.^30^ Additionally, since it is expected that future trials will expand their focus to pediatric populations, evaluating the views of the pediatric patients and their caregivers on clinical trial design in the near future will be essential.^31,32^

In conclusion, the overall experience was positive and none of the participants would advise against trial participation. This study resulted in valuable key points to take into account for patients, sites and sponsors. Additional qualitative or quantitative / prospective studies in other geographic regions and patient populations are necessary to optimize the trial design according to the patient's views and experience.

## Supplemental Material

sj-docx-1-jnd-10.1177_22143602241313117 - Supplemental material for The participants’ perspective on facioscapulohumeral muscular dystrophy trials in The Netherlands – A qualitative study Supplemental material, sj-docx-1-jnd-10.1177_22143602241313117 for The participants’ perspective on facioscapulohumeral muscular dystrophy trials in The Netherlands – A qualitative study by Lizan Stinissen, Joost Kools, Sietse Bouma, Emma Lenssen, Eline Sanders, Anke Lanser, Ria de Haas, Baziel GM van Engelen, Wija Oortwijn and Nicol C Voermans in Journal of Neuromuscular Diseases

sj-docx-2-jnd-10.1177_22143602241313117 - Supplemental material for The participants’ perspective on facioscapulohumeral muscular dystrophy trials in The Netherlands – A qualitative study Supplemental material, sj-docx-2-jnd-10.1177_22143602241313117 for The participants’ perspective on facioscapulohumeral muscular dystrophy trials in The Netherlands – A qualitative study by Lizan Stinissen, Joost Kools, Sietse Bouma, Emma Lenssen, Eline Sanders, Anke Lanser, Ria de Haas, Baziel GM van Engelen, Wija Oortwijn and Nicol C Voermans in Journal of Neuromuscular Diseases

sj-pdf-3-jnd-10.1177_22143602241313117 - Supplemental material for The participants’ perspective on facioscapulohumeral muscular dystrophy trials in The Netherlands – A qualitative study Supplemental material, sj-pdf-3-jnd-10.1177_22143602241313117 for The participants’ perspective on facioscapulohumeral muscular dystrophy trials in The Netherlands – A qualitative study by Lizan Stinissen, Joost Kools, Sietse Bouma, Emma Lenssen, Eline Sanders, Anke Lanser, Ria de Haas, Baziel GM van Engelen, Wija Oortwijn and Nicol C Voermans in Journal of Neuromuscular Diseases
