# Supplementary material for: The participants’ perspective on facioscapulohumeral muscular dystrophy trials in The Netherlands – A qualitative study
Source: J Neuromuscul Dis. 2025 Mar 4;12(3):382–93. doi: 10.1177/22143602241313117 (PMC13142829; doi:10.1177/22143602241313117)
Supplement: sj-docx-1-jnd-10.1177_22143602241313117 - Supplemental material for The participants’ perspective on facioscapulohumeral muscular dystrophy trials in The Netherlands – A qualitative study [file sj-docx-1-jnd-10.1177_22143602241313117.docx]

**Supplemental Table 1: Total number of quotes for the different themes**

| **Themes** | **Number of quotes** |
| --- | --- |
| **Motivation for participation** | **69** |
|  |  |
| **Expectations** | **136** |
| Expectations and hope of the study drug | 101 |
| Adverse events and other concerns | 35 |
|  |  |
| **Trial participation** | **461** |
| General trial experience and study visits | 163 |
| Study drug | 55 |
| Trial information and informed consent | 80 |
| Communication and trust | 163 |
|  |  |
| **Recommendations of participants** | **52** |
| Recommendations for other patients | 13 |
| Recommendations for the sponsor | 12 |
| Recommendations for the study team | 27 |
|  |  |
| **Total** | **718** |
