# Supplementary material for: The participants’ perspective on facioscapulohumeral muscular dystrophy trials in The Netherlands – A qualitative study
Source: J Neuromuscul Dis. 2025 Mar 4;12(3):382–93. doi: 10.1177/22143602241313117 (PMC13142829; doi:10.1177/22143602241313117)
Supplement: sj-docx-2-jnd-10.1177_22143602241313117 - Supplemental material for The participants’ perspective on facioscapulohumeral muscular dystrophy trials in The Netherlands – A qualitative study [file sj-docx-2-jnd-10.1177_22143602241313117.docx]

**Supplemental data 2: Interview guide**

**Introduction:**

Good morning/afternoon. I would like to start by expressing to you my sincere gratitude for taking the time for this interview. Before we begin, I will tell you something about myself. My name is Sietse Bouma, and I am a social researcher at Radboud University, where I am pursuing a Master’s degree in Neurobiology, with the specialization track “Science in Society.” This program trains students from diverse backgrounds to conduct social scientific research and to draw connections between society and science. This means that we have many conversations with people who are active in these areas. Today, I will be having a conversation with you about the FSHD Phase II or III trial you are participating in (or have participated in), as this is an important example of science coming into direct contact with society. As a researcher at Radboud University, I would like to emphasize that I am here as an independent interviewer, and I have not been involved in the trial in which you participated. We have been in contact about this interview and, as stated in the invitation letter, you are aware that this interview will take about one hour and that it will be recorded for the study. In the processing of the data, you will be assigned a study number, and the data will be processed anonymously. Are you still okay with this?

If yes, Start recording

First, I would like to ask you a few brief questions:

Please introduce yourself for the recording by stating your name and age. Additionally, please answer the following questions:

- How long ago were you diagnosed with FSHD?
- How did you become aware of the trial?
- Finally, exactly which trial did you participate in?

The interview will consist of four different sections focusing on:

- Motivation and informed consent
- Communication and interpretation
- Expectations and trust
- Recommendations for future trials

Are you ready to start the interview?

If yes, start interview

**Motivation and informed consent:**

1. What were your main reasons for participating in this clinical genetic study/trial?

- Was there a specific reason that weighed most heavily and determined your decision? If so, what was it?
- Did you have any doubts before deciding to participate in the trial?
  - If so, what made you decide to participate anyway?

1. How was the screening period? (same questions as for informed consent)

*The next few questions are about the informed consent process. Informed consent is the procedure in which you receive all the details about the research setup from the doctor, including the associated advantages and disadvantages. You might recall such a meeting prior to officially participating in the trial.*

If so, I would like to ask you how this informed consent process went.

1. How was the information presented during the informed consent?

- How long did this meeting last?
- Who were present?
- How was the information presented? (written/ oral)

1. Which parts were easy to understand/follow and which parts were less so?

- Was there an opportunity to ask questions?
  - If so, did you take advantage of it?

How did you feel about the opportunity to share the information you received with your own doctor or confidant before agreeing to participate? Was this opportunity clear?

**Communication and interpretation**

- What was your experience with communication during the various phases of the clinical trial or study? By “different phases,” I mean the following moments, which we will now go through briefly. At this point, you can mention any pleasant and unpleasant experiences you can remember.
  - Communication by mail or email in preparation for study visits
  - Direct contact with the research nurse and doctor during the study (during study visits in the hospital)
  - Direct or indirect contact with the corresponding research organization about the progression of the study
  - Contact with the patient organization(s) during and after the trial
- Was it possible to exchange experiences or other news about the trial with other members of the patient organization?
  - If so, did you take advantage of this?
  - If so, what was your experience with it?
  - If not, what might you have thought of this possible addition?
- Was there an opportunity to share personal concerns about the trial during the study?
  - If so, did you take advantage of it?
  - If so, how was it arranged?
- Were you able to ask questions about the trial at any time during the study?
  - At each contact, were you asked if everything was clear/if you had any questions?
  - Were you able to contact the investigators on your own at any time?
  - How did you find that?

**Expectations & trust: *Introduction to expectations***

- What were your expectations of the clinical study/trial prior to participation?
  - How did you think the effects of the therapy would be noticeable?
  - What were your expectations about possible side effects?
  - How did you anticipate the study days and testing?
- To what extent did the clinical study or trial meet the expectations we just discussed? Please elaborate.
- What expectations were not met, and do you know why?

*Introduction to trust*

- Did your level of trust change during the study?
  - If so, to what extent, better or worse?
- How did you experience the results/announcement of results?
  - *Additional follow-up question*
- What did the study or trial mean to you or others you spoke to who also had progressive disease?
  - *In response to answer:* Did you experience this as a positive impact?
- Question exclusively for Phase III patients: Did the success of Phase II affect your expectations/thoughts about the Phase III trial?

**Recommendations**

- Would you recommend others to participate in a clinical trial or study for FSHD? Please explain.
- What are your recommendations for future studies and trials related to FSHD?
  - In what ways could communication by/with physicians or others who are involved be improved?
  - In what way could communication about trial progress and results be improved in the future?

Optional (only if time remains): Participation in the study

- How did the clinical trial or participation in the study affect your daily activities?
  - School
  - Work
  - Exercise
  - Social activities
- Has participation affected any major decisions (such as the desire to have children)?

**Possible answers that can be identified in Question 1:**

- Hope for potential positive change in disease state
- Prevention of progression
- Extension of life span
- The desire to help others
- The will to help science
- No other choice/desperation
